# Chronic widespread bodily pain is increased among individuals with history of fracture: findings from UK Biobank

**DOI:** 10.1007/s11657-015-0252-1

**Published:** 2015-12-17

**Authors:** Karen Walker-Bone, Nicholas C. Harvey, Georgia Ntani, Tannaze Tinati, Gareth T. Jones, Blair H. Smith, Gary J. Macfarlane, Cyrus Cooper

**Affiliations:** 1 MRC Lifecourse Epidemiology Unit, University of Southampton, Southampton, SO16 6YD UK; 2 NIHR Southampton Biomedical Research Centre, University of Southampton and University Hospital Southampton NHS Foundation Trust, Southampton, UK; 3 Musculoskeletal Research Collaboration (Epidemiology Group), University of Aberdeen, Aberdeen, UK; 4 Division of Population Health Sciences, University of Dundee, Dundee, UK; 5 NIHR Musculoskeletal Biomedical Research Unit, University of Oxford, Oxford, UK

**Keywords:** Epidemiology, Chronic widespread pain, Fracture, UK Biobank, Stressors

## Abstract

***Summary*:**

In this cross-sectional analysis of the UK Biobank cohort, a history of fracture was associated with increased risk of current widespread chronic pain.

**Purpose/Introduction:**

We aimed to test the hypothesis that a history of fracture is associated with reporting chronic widespread bodily pain (CWBP), using baseline data from the UK Biobank cohort, comprising 502,656 people aged 40–69 years.

**Methods:**

The case definition of current chronic widespread bodily pain was based on a response of ‘yes’ to the question ‘do you have pain all over the body?’ and ‘yes’ to ‘and have you experienced pain all over the body for more than 3 months?’ Multivariable Poisson regression with robust standard errors was used to test the relationship between fracture (occurring within 5 years prior to the baseline interview, and recorded by self-report) at the spine, hip, upper limb or lower limb and CWBP, adjusting for confounders.

**Results:**

Of 501,733 participants (mean age 56.5 years), 7130 individuals reported CWBP and 23,177 had a history of fracture affecting the upper limb, lower limb, spine and/or hip. Individuals with prior fracture were significantly more likely to report CWBP than those without. After adjustment for potential risk factors (age, gender, demographic, lifestyle and socioeconomic, and psychological), risk ratios were attenuated but remained statistically significant with a more than doubling of risk for CWBP with spine fractures in men (risk ratio (RR) 2.67, 95 % confidence interval (CI) 1.66–4.31; *p* < 0.001) and women (RR 2.13, 95 % CI 1.35–3.37, *p* = 0.001) and with hip fractures in women (RR 2.19, 95 % CI 1.33–3.59; *p* = 0.002).

**Conclusions:**

In this cross-sectional analysis, previous fracture is associated with an increased likelihood of chronic widespread bodily pain, particularly with hip fractures in women, and spine fractures in both sexes. If replicated, these findings may help inform the identification of those most at risk of chronic widespread pain post-fracture, allowing preventative measures to be targeted.

## Background

Chronic widespread pain is common and associated with distress, disability and substantial direct and indirect health care costs [1, 2]. Estimates of prevalence rates vary from 4.7 % [3] to 24 % [4] depending on study design and case definition [2]. Chronic widespread pain became conceptualised in 1990, for the first time within the case definition of a disease, by the American College of Rheumatologists when they defined it as pain that is bilateral, above and below the waist and in the axial skeleton, for more than 3 months. In the presence of an elevated number (11 of 18) of ‘tender points’, as evidence of allodynia, these became the diagnostic criteria for fibromyalgia syndrome [5]. Chronic widespread pain, using this and other definitions [3], has been much studied, and the importance of chronic widespread pain to individuals, health care providers and society as a whole has become widely recognised. It is associated not only with high levels of morbidity but also with increased rates of mortality, attributable mostly to excess cardiovascular disease and cancer [6–8].

A wide variety of exposures have been identified as risk factors for chronic widespread pain, relating not only to social and psychological factors but also to physical insults such as acute ‘whiplash’ injury in road traffic accidents [2]. However, it is unclear whether the occurrence of a bony fracture, a common clinical event causing acute stress with tissue damage, predisposes to an increased risk of chronic widespread pain. Therefore, the aim of the current study was to determine whether fractures were associated with chronic widespread pain, using data from the UK Biobank cohort.

## Methods

### Study subjects

We conducted a cross-sectional analysis using baseline data collected in the UK Biobank study. UK National Health Service (NHS) registers maintain records of almost everybody in the general population (that is, excluding the small number of individuals not legally registered as resident). The protocol is available publicly (http://www.ukbiobank.ac.uk/wp-content/uploads/2011/11/UK-Biobank-Protocol.pdf?phpMyAdmin=trmKQlYdjjnQIgJ%2CfAzikMhEnx6). Using these records, around 9.2 million primary invitations were sent to individuals aged 40–69 years living within a reasonable travelling distance of a total of 22 assessment centres across Great Britain 2007–2010 [9, 10]. This age range was chosen to allow time for a wide range of incident disease events to accrue, permitting case–control studies to be undertaken in the investigation of the determinants of chronic non-communicable diseases of middle and later life.

### Data collection

Participants completed a series of touchscreen computer-based questionnaires followed by a face-to-face interview with trained research staff. Details of the assessments and variables are publicly available (http://biobank.ctsu.ox.ac.uk/crystal/), and a transcript of the touchscreen questionnaire may be downloaded (http://www.ukbiobank.ac.uk/wp-content/uploads/2011/06/Touch_screen_questionnaire.pdf?phpMyAdmin=trmKQlYdjjnQIgJ%2CfAzikMhEnx6). The information collected included socio-demographics (age, gender, ethnicity, educational attainment, employment status, household income, and postcode of residence with corresponding deprivation index score), lifestyle factors (including cigarette smoking, diet, physical activity, and alcohol use), and self-reported history of physician-diagnosed major depression. Participants also completed a series of questions, using a self-administered touchscreen questionnaire, which assessed current symptoms of psychological well-being, and included questions from which a neuroticism score could be calculated, together with indices of social support and social functioning. There were a number of questions about pain in UK Biobank, and for this study, the case definition of current chronic widespread bodily pain (CWBP) was based on a response of ‘yes’ to the question ‘do you have pain all over the body?’ and yes to ‘and have you experienced pain all over the body for more than 3 months?’.

All participants were asked if they had broken/fractured any bone in the previous 5 years and, if they responded positively, they were asked to identify where they had fractured from the following list of sites: ankle, leg, hip, spine, wrist, arm, other or unknown. For the purposes of the current analyses, fractures reported to have occurred in the arm/wrist were amalgamated as ‘upper limb’ fractures and those in the leg/ankle were combined as ‘lower limb’ fractures. Height and weight were measured in all participants by trained data collectors during the clinic attendance using standard operating procedures, and body mass index (BMI) subsequently calculated (kg/m^2^). The Townsend Deprivation Index was used as a measure of socio-economic status (with a greater value corresponding to greater deprivation). This integrates measures of unemployment, non-car ownership, non-home ownership and household overcrowding by neighbourhood across the UK [11].

This study was conducted under generic approval from the NHS National Research Ethics Service (17th June 2011, Ref 11/NW/0382). Participants provided electronic consent for the baseline assessments.

### Statistical analyses

Data were analysed using Stata v12.1 (Statacorp, College Station, TX, USA). We examined the associations between fracture at the four different sites and chronic widespread bodily pain (CWBP), as the outcome, using multivariable Poisson regression models with robust standard errors. In order to explore the modifying effects of anthropometry, lifestyle and psychological measures, we used three incremental multivariate models. The considered covariates are based on their associations (either previously documented or hypothesised) with fracture and chronic pain [2, 12]. In the absence of a specific marker of dietary quality, we used intake of fruit and vegetables, as this has been previously demonstrated to correlate well with quality of the overall diet [13]. Thus associations were first adjusted for age, sex, BMI, and ethnicity (model 1) and were further adjusted for cigarette smoking, alcohol consumption, fruit and vegetable intake, deprivation index, household income and physical activity (model 2). In model 3, we additionally adjusted for neuroticism score, social support and history of major depression.

## Results

### Characteristics of participants

In total, UK Biobank recruited 502,656 individuals. Complete data on CWBP and fracture were available for 501,733 participants, mean age 56.5 years, amongst whom there were 228,724 men and 273,009 women. Most (94.24 %) of the population were Caucasian. A total of 47,475 (9.46 %) participants reported a history of at least one fracture of the upper limb, lower limb, spine or hip over the preceding 5 years. Upper limb fractures were the most common, followed by lower limb, spine and then hip. Amongst those who had sustained fractures, 23,177 (48.8 %) reported fracture at one site, 1219 (2.57 %) reported fractures at two of the sites, 73 (0.15 %) at three sites and 3 (0.01 %) at all four sites. Women reported fractures more commonly than men (6.13 % women vs. 3.99 % men, *p* < 0.001), and Caucasians reported fractures more commonly than all other ethnic groups (4.95 % Caucasians vs. 3.87 % Asian vs. 3.10 % Black, *p* < 0.001). Participants with fractures had a higher median deprivation index than those without (−1.87 vs −2.15, *p* < 0.001). In total, CWBP was reported by 7130 participants (1.42 % overall; prevalence 1.13 % in men and 1.66 % in women). Of the 7130 participants with widespread pain, 986 (13.8 %) had a previous fracture and 580 (8.1 %) had at least one of hip, spine, upper or lower limb fracture. Sixty-five (0.9 %) reported previous fractures at two or more of these sites. The characteristics of those with and without CWBP are summarised in Table 1. Current cigarette smokers reported CWBP more than never smokers (2.48 vs. 1.23 %, *p* < 0.001), and median deprivation index was higher among those with CWBP compared to those without (−0.31 vs. −2.16, *p* < 0.001).

**Table 1:** Characteristics of study participants according to presence of chronic widespread bodily pain (CWBP)

	All	With CWBP	Without CWBP
	*N* = 501,733	*N* = 7130	N = 494,603
Demographic characteristics			
Age (mean (SD))	56.53 (8.09)	57.07 (7.58)	56.52 (8.10)
BMI (mean (SD))	27.43 (4.80)	29.94 (6.10)	27.40 (4.77)
Gender (*n* (%))			
Male	228,724 (45.59)	2586 (36.27)	226,138 (45.72)
Female	273,009 (54.41)	4544 (63.73)	268,465 (54.28)
Ethnic origin (*n* (%))			
White	472,823 (94.2)	6228 (87.35)	466,595 (94.34)
Mixed	2958 (0.59)	82 (1.15)	2876 (0.58)
Asian or Asian British	9877 (1.97)	367 (5.15)	9510 (1.92)
Black or Black British	8063 (1.61)	261 (3.66)	7802 (1.58)
Chinese	1573 (0.31)	14 (0.20)	1559 (0.32)
Other	4560 (0.91)	140 (1.96)	4420 (0.89)
Not known	1879 (0.37)	38 (0.53)	1841 (0.37)
Lifestyle factors			
Smoking (*n* (%))			
Never	273,548 (54.52)	3357 (47.08)	270,191 (54.63)
Ex	173,100 (34.50)	2405 (33.73)	170,695 (34.51)
Current	52,979 (10.56)	1316 (18.46)	51,663 (10.45)
Not known	2106 (0.42)	52 (0.73)	2054 (0.42)
Alcohol (*n* (%))			
Never	40,657 (8.10)	1616 (22.66)	39,041 (7.89)
Special occasions only	58,029 (11.57)	1560 (21.88)	56,469 (11.42)
1–3 times a month	55,871 (11.14)	847 (11.88)	55,024 (11.12)
1–2 times a week	129,321 (25.77)	1485 (20.83)	127,836 (25.85)
3–4 times a week	115,460 (23.01)	842 (11.81)	114,618 (23.17)
Daily or almost daily	101,790 (20.29)	767 (10.76)	101,023 (20.43)
Not known	605 (0.12)	13 (0.18)	592 (0.12)
Physical activity (days a week) (median (IQR))	3 (2–5)	3 (0–5)	3 (2–5)
Fruit (pieces/day) (median (IQR))	2 (1–3)	2 (1–3)	2 (1–3)
Vegetables (tablespoons/day) (median (IQR))	4 (3–6)	4 (3–6)	4 (3–6)
Socio-economic factors			
Deprivation index (median (IQR))	−2.14 (−3.64 to 0.54)	−0.31 (−2.78 to 3.05)	−2.16 (−3.65 to 0.51)
Income (*n* (%))			
<£18,000	97,202 (19.37)	2763 (38.75)	94,439 (19.09)
£18,000–£30,000	108,201 (21.57)	1310 (18.37)	106,891 (21.61)
£31,000–£51,000	110,790 (22.08)	900 (12.62)	109,890 (22.22)
£52,000–£100,000	86,294 (17.20)	424 (5.95)	85,870 (17.36)
>£100,000	22,934 (4.57)	62 (0.87)	22,872 (4.62)
Not known	76,312 (15.21)	1671 (23.44)	74,641 (15.09)
Psychological factors			
Neuroticism (median (IQR))	4 (1–6)	4 (3–9)	4 (1–6)
Major depression (*n* (%))			
No	452,920 (90.27)	5390 (75.60)	447,530 (90.48)
Yes	15,532 (3.10)	979 (13.73)	14,553 (2.94)
Not known	33,281 (6.63)	761 (10.67)	32,520 (6.57)
Social support variables			
Number of social activities			
1	218,701 (43.59)	2910 (40.81)	215,791 (43.63)
2+	128,907 (25.69)	1084 (15.20)	127,823 (25.84)
NA	154,125 (30.72)	3136 (43.98)	150,989 (30.53)
Frequency of friends/family visits			
Weekly	386,270 (76.99)	5442 (76.33)	380,828 (77.00)
Once per month/2 months	99,689 (19.87)	1212 (17.00)	98,477 (19.91)
Never or almost never/no friends/family	8718 (1.74)	333 (4.67)	8385 (1.70)
NA	7056 (1.41)	1.43 (2.01)	6913 (1.40)
Able to confide			
Weekly	358,759 (71.50)	4447 (62.37)	354,312 (71.64)
Once per month/2 months	53,254 (10.61)	794 (11.14)	52,460 (10.61)
Never or almost never	71,750 (14.3)	1517 (21.28)	70,233 (14.20)
NA	17,970 (3.58)	372 (5.22)	17,598 (3.56)

All *p* values for difference between those with and those without CWBP are <0.001, except fruit consumption (*p* = 0.827) and vegetable consumption (*p* = 0.265)

*SD* standard deviation, *BMI* body mass index, *IQR* interquartile range, *NA* not available

### Fracture and risk of CWBP

Table 2 summarises the association between fracture at any of the four sites and current reporting of CWBP. Previous fracture at any anatomical site was associated with increased risk of CWBP (*p* ≤ 0.001). The prevalence of CWBP amongst those reporting a past hip fracture was 4.07 %. The figure for those with past spine fracture was 4.45 % and for those with hip or spine fracture 4.05 %.

**Table 2:** Prevalence of site-specific previous fractures

	All	Without CWBP	With CWBP	
	*N* (%)	*N* (%)	*N* (%)	*p*
Hip				
No	500,849 (99.82 %)	493,755 (99.83 %)	7094 (99.50 %)	<0.001
Yes	884 (0.18 %)	848 (0.17 %)	36 (0.50 %)	
Spine				
No	500,609 (99.78 %)	493,529 (99.78 %)	7080 (99.30 %)	<0.001
Yes	1124 (0.22 %)	1074 (0.22 %)	50 (0.70 %)	
Upper limb (arm or wrist)				
No	487,628 (97.19 %)	480,803 (97.21 %)	6825 (95.72 %)	<0.001
Yes	14,105 (2.81 %)	13,800 (2.79 %)	305 (4.28 %)	
Lower limb (leg or ankle)				
No	492,000 (98.06 %)	485,136 (98.09 %)	6864 (96.27 %)	<0.001
Yes	9733 (1.94 %)	9467 (1.91 %)	266 (3.73 %)	
Other				
No	478,730 (95.42)	472,006 (95.43)	6724 (94.31)	<0.001
Yes	406 (4.58)	22,597 (4.57)	406 (5.69)	
	Women	Without CWBP	With CWBP	
	*N* (%)	*N* (%)	*N* (%)	*p*
Hip				
No	272,507 (99.82 %)	267,985 (99.82 %)	4522 (99.52 %)	<0.001
Yes	502 (0.18 %)	480 (0.18 %)	22 (0.48 %)	
Spine				
No	272,364 (99.76 %)	267,844 (99.77 %)	4520 (99.47 %)	<0.001
Yes	645 (0.24 %)	621 (0.23 %)	24 (0.53 %)	
Upper limb (arm or wrist)				
No	263,437 (96.49 %)	259,093 (96.51 %)	4344 (95.60 %)	0.001
Yes	9572 (3.51 %)	9372 (3.49 %)	200 (4.40 %)	
Lower limb (leg or ankle)				
No	267,004 (97.80 %)	262,628 (97.83 %)	4376 (96.30 %)	<0.001
Yes	6005 (2.20 %)	5837 (2.17 %)	168 (3.70 %)	
Other				
No	260,991 (95.60)	256,706 (95.62)	4285 (94.30)	<0.001
Yes	12,018 (4.40)	11,759 (4.38)	259 (5.70)	
	Men	Without CWBP	With CWBP	
	*N* (%)	*N* (%)	*N* (%)	*p*
Hip				
No	228,342 (99.83 %)	225,770 (99.84 %)	2572 (99.46 %)	<0.001
Yes	382 (0.17 %)	368 (0.16 %)	14 (0.54 %)	
Spine				
No	228,245 (99.79 %)	225,685 (99.80 %)	2560 (98.99 %)	<0.001
Yes	479 (0.21 %)	453 (0.20 %)	26 (1.01 %)	
Upper limb (arm or wrist)				
No	224,191 (98.02 %)	221,710 (98.04 %)	2481 (95.94 %)	<0.001
Yes	4533 (1.98 %)	4428 (1.96 %)	105 (4.06 %)	
Lower limb (leg or ankle)				
No	224,996 (98.37 %)	222,508 (98.39 %)	2488 (96.21 %)	<0.001
Yes	3728 (1.63 %)	3630 (1.61 %)	98 (3.79 %)	
Other				
No	217,739 (95.20)	215,300 (95.21)	2439 (94.32)	0.035
Yes	10,985 (4.80)	10,838 (4.79)	147 (5.68)	

*CWBP* chronic widespread bodily pain

**Fig. 1:**
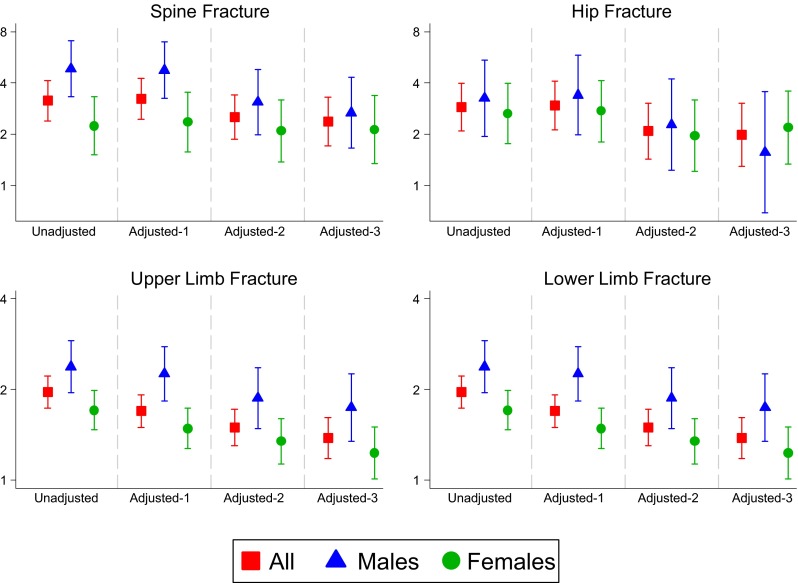
Risk estimates for CWBP from previous fracture at each site in unadjusted models and then after adjustment for potential confounding factors. Adjusted 1: Adjusted for sex, age, BMI, ethnicity; Adjusted 2: As for Adjusted 1 and further adjusted for smoking, alcohol, fruit consumption, vegetables consumption, physical activity, deprivation index and income; Adjusted 3: As for Adjusted 2 and further adjusted for neuroticism score and major depression

In the overall Poisson regression analyses, adjusted for age, sex, BMI and ethnicity (model 1), there were significant associations between CWBP and fracture at all sites (all *p* < 0.001, Table 3). Having a prior fracture was significantly associated with CWBP, and the rate ratios (RRs) ranged from 1.29 for females with upper limb fractures through to 4.75 for males with spine fractures. After further adjustment for lifestyle factors (model 2) (smoking, alcohol, fruit and vegetable intake, physical activity, household income and deprivation index), significant associations remained between CWBP and fracture for all sites (all *p* < 0.05) except the upper limb in women. However, there was slight attenuation of the RRs (lowest 1.35 for females with lower limb fractures through to 3.09 for males with spine fractures). In model 3, additional adjustment for psychological factors (major depression, social support and neuroticism score) further attenuated the RRs, but strongly statistically significant associations remained with greater than a doubling of risk of CWBP in women with spine (RR 2.13; 95 % confidence interval (CI) 1.35 to 3.37) and hip fractures (RR 2.19; 95 % CI 1.33 to 3.59) and in men with spine fractures (RR 2.67; 95 % CI 1.66 to 4.31; Fig. 1).

**Table 3:** Associations between CWBP and site-specific previous fractures

Fracture sites						
	All		Females		Males	
	RR (95 % CI)	*p*	RR (95 % CI)	*p*	RR (95 % CI)	*p*
Hip						
Unadjusted	2.88 (2.09, 3.96)	<0.001	2.64 (1.75, 3.98)	<0.001	3.25 (1.94, 5.45)	<0.001
Adjusted 1	2.95 (2.12, 4.10)	<0.001	2.73 (1.80, 4.13)	<0.001	3.39 (1.98, 5.81)	<0.001
Adjusted 2	2.08 (1.43, 3.04)	<0.001	1.96 (1.21, 3.17)	0.006	2.28 (1.23, 4.22)	0.009
Adjusted 3	1.98 (1.30, 3.03)	0.002	2.19 (1.33, 3.59)	0.002	1.57 (0.69, 3.55)	0.282
Spine						
Unadjusted	3.15 (2.40, 4.13)	<0.001	2.24 (1.51, 3.32)	<0.001	4.84 (3.32, 7.05)	<0.001
Adjusted 1	3.22 (2.44, 4.24)	<0.001	2.35 (1.57, 3.52)	<0.001	4.75 (3.24, 6.96)	<0.001
Adjusted 2	2.52 (1.86, 3.41)	<0.001	2.09 (1.37, 3.19)	0.001	3.09 (1.99, 4.80)	<0.001
Adjusted 3	2.37 (1.70, 3.31)	<0.001	2.13 (1.35, 3.37)	0.001	2.67 (1.66, 4.31)	<0.001
Upper limb (arm or wrist)						
Unadjusted	1.54 (1.38, 1.73)	<0.001	1.27 (1.10, 1.46)	0.001	2.09 (1.73, 2.54)	<0.001
Adjusted 1	1.48 (1.32, 1.67)	<0.001	1.29 (1.12, 1.48)	<0.001	2.12 (1.74, 2.57)	<0.001
Adjusted 2	1.31 (1.15, 1.49)	<0.001	1.13 (0.96, 1.32)	0.145	1.91 (1.54, 2.37)	<0.001
Adjusted 3	1.24 (1.07, 1.43)	0.004	1.11 (0.93, 1.33)	0.238	1.63 (1.27, 2.09)	<0.001
Lower limb (leg or ankle)						
Unadjusted	1.96 (1.74, 2.21)	<0.001	1.71 (1.47, 1.99)	<0.001	2.38 (1.95, 2.90)	<0.001
Adjusted 1	1.69 (1.50, 1.92)	<0.001	1.48 (1.27, 1.73)	<0.001	2.25 (1.83, 2.77)	<0.001
Adjusted 2	1.49 (1.30, 1.72)	<0.001	1.35 (1.13, 1.60)	0.001	1.87 (1.48, 2.36)	<0.001
Adjusted 3	1.38 (1.18, 1.61)	<0.001	1.23 (1.01, 1.50)	0.039	1.74 (1.35, 2.26)	<0.001
Other						
Unadjusted	1.26 (1.14, 1.39)	<0.001	1.31 (1.16, 1.49)	<0.001	1.19 (1.01, 1.41)	0.035
Adjusted 1	1.33 (1.21, 1.47)	<0.001	1.35 (1.19, 1.52)	<0.001	1.31 (1.11, 1.55)	0.002
Adjusted 2	1.31 (1.18, 1.46)	<0.001	1.34 (1.17, 1.53)	<0.001	1.27 (1.06, 1.52)	0.011
Adjusted 3	1.25 (1.10, 1.41)	<0.001	1.28 (1.10, 1.48)	0.001	1.20 (0.98, 1.47)	0.084

Adjusted 1: Adjusted for sex, age, BMI, ethnicity; Adjusted 2: As for Adjusted 1 and further adjusted for smoking, alcohol, fruit consumption, vegetables consumption, physical activity, deprivation index and income; Adjusted 3: As for Adjusted 2 and further adjusted for neuroticism score and major depression; RR: risk ratio; CI: confidence interval

## Discussion

Overall, individuals who had experienced a fracture were significantly more likely to also report CWBP than people who did not report a fracture. These effects were observed for fractures at the spine, hip, upper and lower limbs, and other sites but were most pronounced for spine and hip fractures where a doubling of risk was observed, independent of potential confounding factors.

Although a history of fracture has not previously been specifically associated with chronic widespread pain, the characteristics of other traumatic events, which have been linked to this outcome, may aid understanding of our current findings. Stressful events and trauma have been implicated in chronic widespread pain previously [12, 14–17]. For example, rates of chronic widespread pain have been shown to increase immediately after major disasters such as earthquakes and hurricanes with peaks that are bimodal, one occurring immediately after the event (presumably attributable to direct trauma) but with a second peak of pain a few weeks later, less well explained by acute trauma [18]. The relationship between motor vehicle collisions and chronic widespread pain and fibromyalgia has been widely investigated [12, 19–21]. Interestingly, there is evidence that the risk of whiplash syndrome after a vehicle collision is more associated with the pre-morbid health of the individual than the nature of the collision itself [12]; there is a weak relationship between the degree of damage or abnormality in the neck and the degree of pain or functional impairment that an individual experiences [20, 21]. It could be that it is the environment within which an event occurs that may be the most important determinant of the physiological consequences. There is evidence from neuroendocrine studies that it is those stressors perceived by the individual as inescapable or unavoidable, or which are unpredictable or occur when an individual lacks social support, which evoke the strongest adverse biological consequences [22]. Within this construct of causation, it is possible to conjecture that fractures are acute, traumatic uncontrollable life events which could be important in determining risk of chronic widespread pain in a genetically, psychologically or biologically predisposed individual. An alternative explanation would be that chronic widespread pain, possibly through reduced physical activity [3, 23], might predispose to fracture. Conversely, in this age group, where trauma is an important contributor to fracture pathogenesis [24], lowered physical activity might also lead to a reduced exposure to situations in which physical trauma might be experienced. Unfortunately, it was not possible to explore this point further in the present study as information on the level of trauma involved in each fracture was not available.

The mechanisms underlying chronic widespread pain are likely to be complex and are currently poorly understood. There is growing evidence for a role of genetic factors [25], early life effects on neuronal plasticity, central sensitisation [19] and involvement of neuro-hormonal systems such as the hypothalamic-pituitary-adrenal axis [26] and sympathetic nervous system [19]. Undoubtedly, stress (acute and chronic) causes psychological manifestations that are associated with the experience and reporting of pain. It is interesting therefore that, although attenuated, the association between CWBP and fracture remained even after adjustment for psychological factors, particularly for fractures at the hip and spine. It could be that acute or chronic trauma or stress leads to important lifestyle changes which have an effect on tolerance of or perception of pain, for example alterations in physical activity, smoking or alcohol consumption. However, our statistical analyses demonstrated persisting associations between prior fracture and CWBP after adjustment for such factors. Our data suggest that the mechanism cannot be purely psychological or lifestyle-mediated and, therefore, lend some epidemiological support to the hypothesis of Lyon and colleagues who suggested that chronic widespread pain could be part of a whole-organism stress response which is evolutionarily conserved and follows a pattern found in the simplest living systems [27].

Interestingly, the associations appeared strongest for fractures at the hip and spine, compared with fractures in the upper or lower limbs. These latter groupings clearly represent a more heterogeneous range of fractures, some major and some more minor (e.g. wrist) with less impact on quality of life and fewer specific effects likely to lead to chronic pain [24]. In contrast, the high levels of morbidity and decreased survival following a hip and spine fractures is well documented, as are the potential changes in body shape, such as kyphosis, leading to pain and respiratory difficulties following vertebral fracture [24]. The associations also appeared of greater magnitude in men than in women, and it is unclear what might explain this observation, but greater exposure to trauma as a cause of fractures amongst men might be one factor. Finally, although the maximum effect size that we observed represented more than a doubling of risk, given the low prevalence of CWBP in our cohort, the absolute risk difference for CWBP between those with no fracture (prevalence 1.37 %) and hip or spine fractures (prevalence 4.05 %) was 2.68 %. However, given the vast number of fractures annually across the country [28], these figures still represent a significant disease burden overall.

In considering these results, the following limitations are important. The inclusion of large sample sizes can lead to small differences between groups reaching the threshold of statistical significance, which may not in fact reflect a clinically meaningful difference. However, we observed risk ratios around 2, which suggest biologically important relationships. Second, the cross-sectional nature of the data collection for the current study prevents the investigation of the temporal associations between fracture and CWBP. People were reporting current CWBP and a history of fractures, but they may have sustained fractures very recently or many years previously and we are unable to explore this further within the current study. The lack of temporal clarity is likely to have introduced noise into the analyses and therefore biased towards the null hypothesis, and clearly impairs any ability to make causal influences. Furthermore, onset of CWBP may have been prior to the fracture event. Additionally, the age range of participants was relatively narrow, with older patients, who will be at higher fracture risk, under-represented. Although this does affect the generalisability of our findings, there is no reason to suppose that it would have influenced the results within the cohort. Third, it was not possible to use the definition of chronic widespread pain proposed by the American College of Rheumatologists, and thus we could not compare case definitions. This is also likely to contribute to the low prevalence of our definition in the cohort. Furthermore, as with many such studies, it is possible that there was a healthy selection bias in those individuals willing to take part, which again reduces generalisability, but, if anything, would tend to bias towards the null hypothesis. Fourth, the fracture data analysed here are self-reported and there is no possibility of verifying the fractures from clinical databases or radiographic analyses. It is possible that people with CWBP recall fractures more readily than people without CWBP [29], but the pain and fracture questions were a small part of a large battery of questions, with pain/ fracture not cited as one of the major outcomes of interest of the UK Biobank study. It is unlikely that recall bias could explain differential relationships by fracture site, but it remains possible that recall bias may have contributed to the associations observed overall. Finally, the specific diagnosis of osteoarthritis is not captured at individual joints in UK Biobank and, thus, it was not possible to explore whether osteoarthritis might account for some of the reports of CWBP.

In summary, these results suggest that a history of fracture is associated with increased likelihood of the presence of CWBP with associations that are not fully explained by psychological factors. These relationships were particularly apparent for hip fracture in women and spine fractures in both sexes. However, as these findings are cross-sectional, and the dates of fracture and onset of chronic pain were not known, replication of these findings in further cohorts will be required, ideally in prospective studies. With such an approach, if the association appears to be causal, these findings may inform the identification of those most at risk of chronic widespread pain post fracture, allowing preventative measures to be targeted and the impact of these debilitating sequelae ameliorated.
